# HOTAIR drives autophagy in midbrain dopaminergic neurons in the substantia nigra compacta in a mouse model of Parkinson’s disease by elevating NPTX2 *via* miR-221-3p binding

**DOI:** 10.18632/aging.103028

**Published:** 2020-05-12

**Authors:** Yue Lang, Yu Li, Haojia Yu, Lulu Lin, Xingchi Chen, Sainan Wang, Hui Zhang

**Affiliations:** 1 Department of Neurology, The Second Hospital of Dalian Medical University, Dalian 116023, P.R. China

**Keywords:** HOX transcript antisense intergenic RNA, microRNA-221-3p, neuronal pentraxin II, autophagy, Parkinson’s disease

## Abstract

Parkinson’s disease (PD) is a neurodegenerative disorder characterized by progressive cell loss, largely confined to mesencephalic dopamine neurons of the substantia nigra. This study investigated the functional relevance of the HOX transcript antisense intergenic RNA (HOTAIR)/microRNA-221-3 (miR-221-3p)/neuronal pentraxin II (NPTX2) axis in the process of dopaminergic neuron autophagy using PD mouse models. The PD mouse models were established by intraperitoneal injection of 1-methyl-4-phenyl-1,2,3,6-tetrahydropyrindine (MPTP), while PD cell model was constructed by pretreatment with 1-methyl-4-phenylpyridinium (MPP^+^). The expression of HOTAIR was then examined using RT-qPCR. In addition, the interactions between HOTAIR, miR-221-3p, and NPTX2 were detected through RIP and dual-luciferase reporter gene assays. CCK-8 assay was performed to measure cell viability, and the expression of autophagy-related genes was determined using Western blot analysis. HOTAIR was found to be significantly expressed in the substantia nigra compact tissues and MN9D cells following PD modeling. HOTAIR could bind to miR-221-3p and elevate the NPTX2 expression, which resulted in diminished cell viability and enhanced autophagy of dopaminergic neurons both *in vitro* and *in vivo*. In summary, down-regulation of HOTAIR could potentially inhibit the autophagy of dopaminergic neurons in the substantia nigra compacta in a mouse model of PD, thus saving the demise of dopaminergic neurons.

## INTRODUCTION

Parkinson’s disease (PD) is a common, progressive nervous system disorder that leads to motor and cognitive disability [1]. The occurrence of PD is correlated with age and primarily afflicts the elderly populace, with a prevalence rate of 2.5% in the 60 – 79 years old population and around 3.5% in the over 80 years old population [2]. The chief motor symptoms of PD include shaking or tremor in the limbs, muscle rigidity, impaired posture and loss of balance [3]. Pathologically, the major change in PD tissues is the evident death of dopaminergic neurons in the substantia nigra compacta (SNc) of brain [3]. Dopaminergic neurons are the main sources of dopamine, a neurotransmitter between neurons which controls the functioning of the brain including muscle control, stress and mood [4]. PD has traditionally been considered a disease of dopaminergic neurons in the substantia nigra [5]. Intriguingly, advancements in research have identified some genetic alterations as high-risk factors for PD. Over 500,000 genetic variants including single nucleotide polymorphisms (SNP) throughout human genome have shown association with PD in large population samples [6]. Moreover, proteins such as SNCA, LRRK2, parkin and GBA have also been linked with the development of PD [7–9]. However, research into noncoding RNAs in relation with PD is at a very early stage.

Long non-coding RNAs (lncRNAs) are functional non-coding RNA over 200 nt long which play important roles in tissue development and disease progression [10]. Brain is by far the most complex tissue in humans and contains the most diverse lncRNAs compared to all other tissues, which suggests that lncRNAs might play critical roles in overall brain function [11]. PD-related lncRNAs were first reported in 2014 [12]. Furthermore, lncRNAs have also recently emerged as a new class of genes that regulate cellular processes [13]. The HOTAIR lncRNA is an oncogene that can possess the ability to promote tumorigenesis in several types of cancers [14]. Accumulating evidence has further indicated that HOTAIR has the capacity to activate autophagy [15], a cellular process capable of contributing to the pathogenesis of PD and thus, possibly representing a new molecular target for treating PD [16]. Moreover, recent studies have illustrated that HOTAIR can aggravate the PD progression [17, 18]. Meanwhile, microRNAs (miRNAs or miRs) are also known to be differentially expressed in PD and highlighted as novel markers in PD diagnosis and therapeutics [19]. For instance, down-regulated serum miR-221 has been identified as a potential biomarker for PD evaluation [20]. The preliminary findings in the current study identified the Neuronal pentraxin II gene (NPTX2) as a target of miR-221-3p using dual-luciferase reporter gene assay. This specific gene encodes secreted pentraxins and regulates excitatory synapse formation. Reduction of NPTX2 has also been correlated with neurodegenerative diseases, such as Alzheimer’s disease [21]. In addition, NPTX2 has been illustrated to be the most up-regulated gene in the Parkinsonian substantia nigra [22]. Based on data from previous studies, we aim to elucidate the possible effects of HOTAIR on the autophagy of midbrain dopaminergic neurons in PD models along with the underlying mechanisms involving miR-221-3p-mediated NPTX2.

## RESULTS

### HOTAIR is highly expressed in PD and participates in the progression of PD

**Figure 1:**
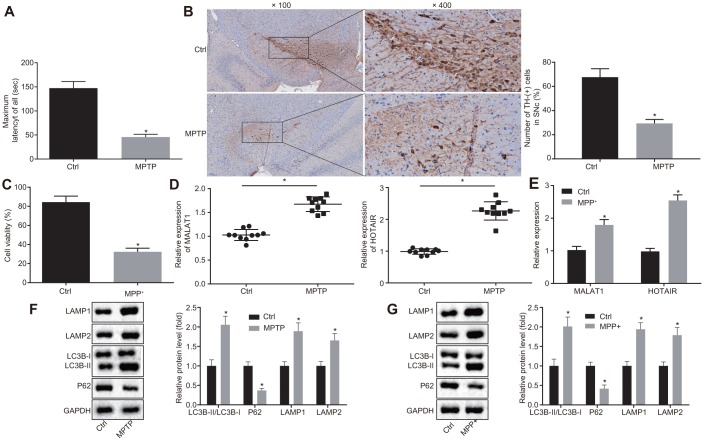
**HOTAIR expression is elevated in PD.** Abnormal motor functions of MPTP-treated and normal saline-treated mice evaluated using the rota rod test. (**A**) The number of TH-positive cells in SNc tissues of mice treated with MPTP or normal saline measured by IHC. (**B**) The viability of MN9D cells undergone MPP+ or PBS treatment detected by CCK-8 assay. (**C**) The expression patterns of MALAT1 and HOTAIR in the SNc tissues of mice treated with MPTP or normal saline determined by RT-qPCR. (**D**) The expression patterns of MALAT1 and HOTAIR in MN9D cells undergone MPP+ or PBS treatment determined by RT-qPCR. (**E**) The ratios of LC3B-I/LC3B-II and LAMP1/LAMP2 along with the P62 expression patterns in SNc tissues of mice treated with MPTP or normal saline determined by Western blot analysis. (**F**) The ratios of LC3B-I/LC3B-II and LAMP1/LAMP2 along with the P62 expression patterns in MN9D cells undergone MPP+ or PBS treatment, as measured by Western blot analysis. (**G**) *p < 0.05 vs. normal saline-treated mice or PBS-treated MN9D cells. Data (mean ± standard deviation) between two groups were compared using unpaired t-test. The experiment was repeated three times. HOTAIR, HOX transcript antisense intergenic RNA; PD, Parkinson’s disease; MPTP, 1-methyl-4-phenyl-1,2,3,6-tetrahydropyrindine; TH, T helper; SNc, substantia nigra compact; IHC, immunohistochemistry; MPP+, 1-methyl-4-phenylpyridinium; PBS, phosphate buffered saline; CCK-8, cell counting kit-8; MALAT1, metastasis associated in lung adenocarcinoma transcript 1; RT-qPCR, reverse transcription quantitative polymerase chain reaction; LC3, light chain 3.

C57BL/6 mice were initially injected with 1-methyl-4-phenyl-1,2,3,6-tetrahydropyrindine (MPTP) to establish PD mice models. Abnormal motor functions of mice were then assessed using the rota rod test. It was found that the MPTP-treated mice spent less time on the rotating rod compared to the normal mice (*p* < 0.05) (Figure 1A). In addition, Immunohistochemistry (IHC) results indicated that there were fewer tyrosine hydroxylase (TH)-positive cells in SNc of mice treated with MPTP in comparison with the SNc of mice treated with normal saline (*p* < 0.05) (Figure 1B), signifying that MPTP-induced PD mice models were successfully established.

Subsequently, the dopaminergic neuronal cell line MN9D was treated with 1-methyl-4-phenylpyridinium (MPP^+^) to induce a cell model of PD *in vitro*. As determined by the cell counting kit-8 (CCK-8) assay, the viability of MN9D cells treated with MPP^+^ was weaker compare to the MN9D cells treated with PBS (*p* < 0.05) (Figure 1C), which was indicative of successful induction of PD cell models *in vitro*.

Additionally, microarray-based analyses were performed to retrieve PD-related lncRNAs. With “Parkinson’s disease” serving as the keyword, 14 PD-related lncRNAs were obtained from the LncRNADisease database, among which 4 lncRNAs (MALAT1, UCHL1-AS1, AK021630, and HOTAIR) were annotated to be implicated in the regulation of PD progression (Supplementary Table 1); however, the murine sequences of UCHL1-AS1 and AK021630 were not available in the array. Hence, reverse transcription quantitative polymerase chain reaction (RT-qPCR) was adopted to detect the expression of MALAT1 and HOTAIR in SNc tissues of mice following MPTP-induced PD modeling. Compared with the SNc tissues of normal saline-treated mice, HOTAIR was found to be up-regulated in the SNc tissues of MPTP-treated mice, and the expression of HOTAIR was much higher than that of MALAT1 (*p* < 0.05) (Figure 1D). Similar results were obtained in cell models of PD *in vitro* (Figure 1E).

Furthermore, Western blot analysis was applied to measure the ratio of light chain 3 (LC3B)-I/LC3B-II, lysosomal-associated membrane protein Type 1 (LAMP1)/LAMP2 as well as the expression patterns of P62 in order to investigate the autophagy of SNc in PD mice. It was demonstrated that the ratio of LC3B-II/LC3B-I and LAMP1/LAMP2 was raised and the expression of P62 was reduced in SNc tissues of PD mice versus that in the SNc tissues of normal saline-treated mice (*p* < 0.05) (Figure 1F). These results were consistent with the trends observed in cell models of PD *in vitro* (*p* < 0.05) (Figure 1G), revealing that MPTP or MPP^+^ induced autophagy in PD.

### HOTAIR silencing suppresses autophagy in dopaminergic neurons in SNc of PD mice

The above results showed that HOTAIR was over-expressed in PD and induced autophagy in PD. Studies have demonstrated the association of HOTAIR expression with autophagy [23–25]. Thus, we conducted the following studies to dissect out the effects of HOTAIR on autophagy in the PD mouse models and cell models.

**Figure 2:**
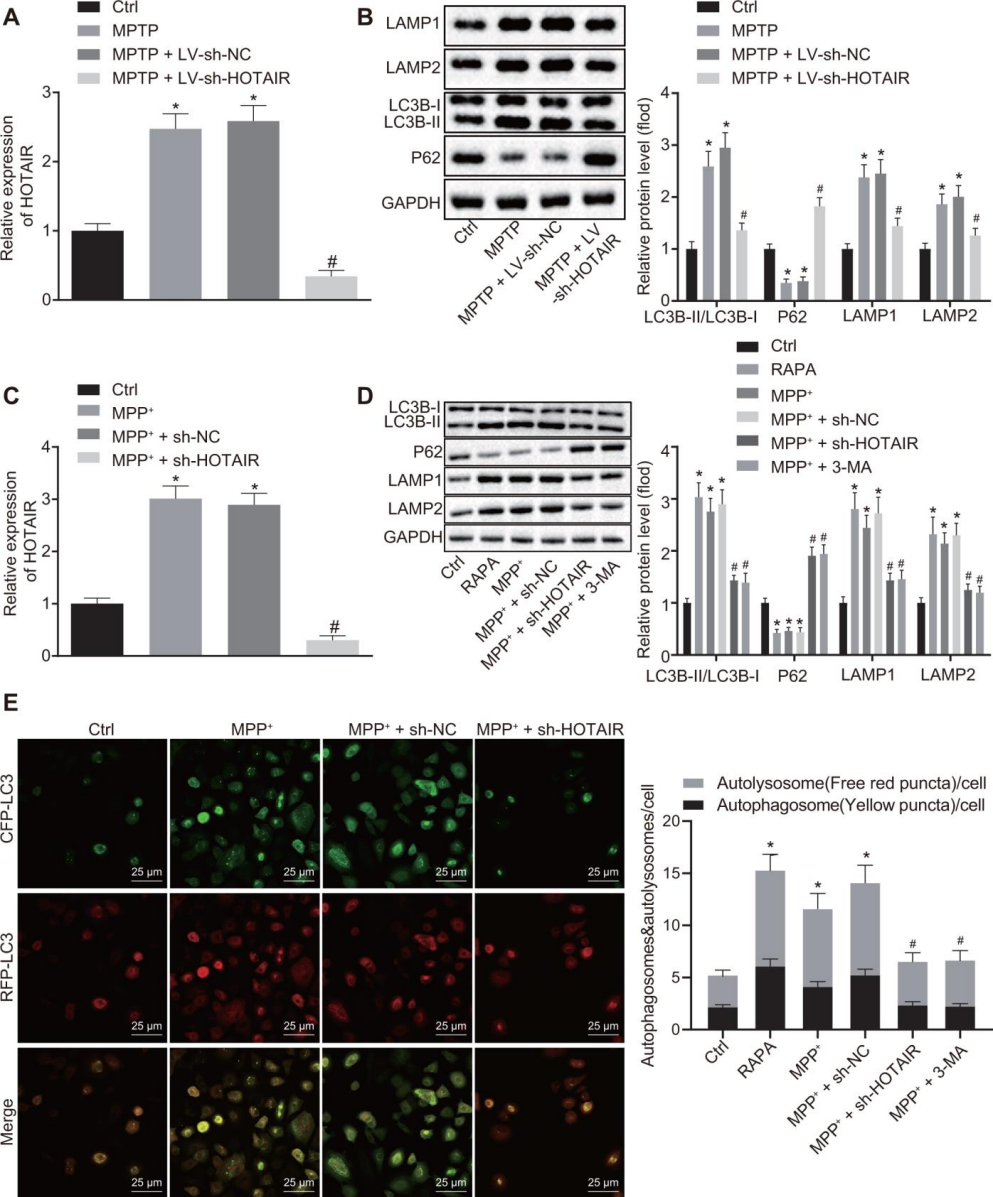
**HOTAIR depletion represses autophagy in dopaminergic neurons in SNc tissues of PD mice.** The expression patterns of HOTAIR in SNc tissues measured by RT-qPCR (n = 10), *p < 0.05 *vs*. mice injected with saline; #*p* < 0.05 *vs*. mice injected with MPTP + LV-sh-NC. (**A**) The ratios of LC3B-I/LC3B-II and LAMP1/LAMP2 along with the P62 expression patterns in SNc tissues determined by Western blot analysis (n = 10), *p < 0.05 *vs*. mice injected with saline; #*p* < 0.05 *vs*. mice injected with MPTP + LV-sh-NC. (**B**) The expression patterns of HOTAIR in MN9D cells treated with PBS, MPP^+^, MPP^+^ + sh-NC and MPP^+^ + sh-HOTAIR measured by RT-qPCR, *p < 0.05 *vs*. MN9D cells treated with PBS; #*p* < 0.05 *vs*. MN9D cells treated with MPP^+^ + sh-NC. (**C**) The ratios of LC3B-I/LC3B-II and LAMP1/LAMP2 along with the P62 expression patterns in MN9D cells treated with PBS, RAPA, MPP^+^, MPP^+^ + sh-NC and MPP^+^ + sh-HOTAIR assessed by Western blot analysis, *p < 0.05 *vs*. MN9D cells treated with PBS; #*p* < 0.05 *vs*. MN9D cells treated with MPP^+^ + sh-NC. (**D**) Detection of autophagy levels in MN9D cells by co-localization analysis. (**E**) Data (mean ± standard deviation) among multiple groups were analyzed using one-way ANOVA and subjected to Tukey’s post-hoc test. The experiment was repeated three times. HOTAIR, HOX transcript antisense intergenic RNA; SNc, substantia nigra compact; PD, Parkinson’s disease; RT-qPCR, reverse transcription quantitative polymerase chain reaction; PBS, phosphate buffered saline; MPP^+^, 1-methyl-4-phenylpyridinium; NC, negative control; ANOVA, analysis of variance; MPTP, 1-methyl-4-phenyl-1,2,3,6-tetrahydropyrindine.

Initially, the expression patterns of HOTAIR, LC3B-I, LC3B-II and P62 in the SNc tissues of grouped mice were detected using RT-qPCR and Western blot analysis. The results demonstrated that the expression of HOTAIR, as well as the ratio of LC3B-II/LC3B-I, and LAMP1/LAMP2 was raised, while the expression of P62 was decreased in mice injected with MPTP and mice injected with MPTP + lentivirus short hairpin-negative control (LV-sh-NC) compared with the mice injected with saline (all *p* < 0.05). Meanwhile, the expressions of HOTAIR and the ratio of LC3B-II/LC3B-I and LAMP1/LAMP2 were lower in mice injected with MPTP + LV-sh-HOTAIR compared to the mice injected with MPTP + LV-sh-NC, while the expression of P62 was higher (all *p* < 0.05) (Figure 2A, 2B).

Subsequently, the expression patterns of HOTAIR and P62, as well as the ratio of LC3B-I/LC3B-II and LAMP1/LAMP2 in MN9D cells were measured using RT-qPCR and Western blot analysis. The results of RT-qPCR revealed that compared with PBS-treated MN9D cells, the expression of HOTAIR was increased in MPP^+^ or MPP^+^ + sh-NC-treated MN9D cells, while being decreased in MN9D cells treated with MPP^+^ + sh-HOTAIR (*p* < 0.05) (Figure 2C). In addition, Western blot analysis results displayed that the ratio of LC3B-II/LC3B-I and LAMP1/LAMP2 was increased, while the expression of P62 was decreased in MN9D cells treated with rapamycin (RAPA), MPP^+^ or MPP^+^ + sh-NC (*p* < 0.05). However, MPP^+^ + sh-HOTAIR treatment resulted in reduced ratios of LC3B-II/LC3B-I and LAMP1/LAMP2 and up-regulated P62 expressions (all *p* < 0.05) (Figure 2D). Additionally, co-localization analysis was performed in cells transfected with mRFP-GFP-LC3 to detect the level of autophagy. It was found that compared with the PBS-treated cells, autophagy levels were significantly increased in cells treated with RAPA, MPP^+^ or MPP^+^ + sh-NC, while that of cells treated with MPP^+^ + sh-HOTAIR and MPP ^+^ + 3-methyladenine (3-MA) was markedly decreased (Figure 2E). These results demonstrated that silencing HOTAIR inhibited autophagy in dopaminergic neurons in SNc tissues of PD mice.

### HOTAIR binds to miR-221-3p

**Figure 3:**
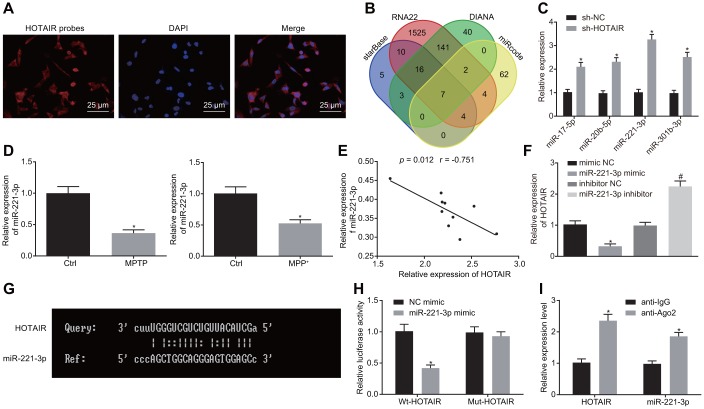
**HOTAIR binds to miR-221-3p and consequently down-regulates its expression.** Subcellular localization of HOTAIR in MN9D cells detected by FISH assay. (**A**) The prediction of downstream miRNAs regulated by HOTAIR in starBase and RNA22 etc.. (**B**) The expression patterns of the 4 screened miRNAs measured using RT-qPCR, *p < 0.05 *vs.* MN9D cells treated with sh-NC. (**C**) The expression patterns of miR-221-3p in the SNc tissues (n = 10) of PD mouse models and PD cell models determined by RT-qPCR, *p < 0.05 *vs.* mice injected with normal saline or cells treated with PBS. (**D**) Pearson’s correlation analysis of the expressions of HOTAIR and miR-221-3p in the SNc tissues of PD mouse models. (**E**) The expression patterns of HOTAIR in PD cell models determined by RT-qPCR, *p < 0.05 *vs*. cells treated with mimic NC. #*p* < 0.05 *vs*. cells treated with inhibitor NC. (**F**) The complementary base paring diagram of HOTAIR and miR-221-3p sequences predicted online. (**G**) The binding of HOTAIR to miR-221-3p measured by dual-luciferase reporter gene assay, *p < 0.05 *vs*. the cells treated with NC mimic. (**H**) The binding of miR-221-3p and HOTAIR assessed by the RIP assay, *p < 0.05 *vs*. cells treated with anti-IgG. (**I**) Data (mean ± standard deviation) between two groups were compared using unpaired *t*-test. The experiment was repeated three times. HOTAIR, HOX transcript antisense intergenic RNA; PD, Parkinson’s disease; miR-221-3p, microRNA-221-3p; FISH, fluorescence in situ hybridization; RT-qPCR, reverse transcription quantitative polymerase chain reaction; SNc, substantia nigra compact; PBS, phosphate buffered saline; wt, wild type; mut, mutase; NC, negative control; RIP, RNA binding protein immunoprecipitation assay.

After uncovering the role of HOTAIR in autophagy in PD, we shifted our focus to the downstream regulatory mechanism of HOTAIR in PD. Fluorescence in situ hybridization (FISH) assay revealed that HOTAIR was primarily expressed in the cytoplasm of TH-positive neurons (Figure 3A). Subsequently, the downstream miRNAs of HOTAIR were predicted in the starBase, RNA22 database etc., with 7 intersection miRNAs (miR-17-5p, miR-20b-5p, miR-4295, miR-217, miR-221-3p, miR-301b-3p, and miR-3666) obtained (Figure 3B). However, the murine sequences of miR-4295, miR-217 and miR-3666 were not available. The expression patterns of miR-17-5p, miR-20b-5p, miR-221-3p, miR-301b-3p in MN9D cells treated with PBS or sh-HOTAIR were then detected using RT-qPCR, which demonstrated that miR-221-3p exerted the highest expression when HOTAIR was silenced (*p* < 0.05) (Figure 3C). Additionally, the expressions of miR-221-3p were further determined in the SNc tissues of PD mouse models and PD cell models, which revealed that miR-221-3p levels were down-regulated in the SNc tissues of PD mouse models and PD cell models respectively compared with the corresponding treatments (all *p* < 0.05) (Figure 3D).

Next, correlation analysis was performed for the expressions of HOTAIR and miR-221-3p, which demonstrated that the expressions of HOTAIR and miR-221-3p were negatively-correlated in 10 SNc tissues of PD mice (Figure 3E). PD cells were further treated with mimic NC, miR-221-3p mimic, inhibitor NC, miR-221-3p inhibitor. HOTAIR expression patterns were then detected in PD cells using RT-qPCR, which verified that the expressions of HOTAIR and miR-221-3p were negatively-correlated (Figure 3F). Moreover, the binding sites between HOTAIR and miR-221-3p were predicted by an online website (Figure 3G), and further confirmed using a dual-luciferase reporter gene assay (Figure 3H): compared with the cells treated with mimic NC, luciferase activity was decreased in miR-221-3p mimic treated cells co-transfected with wild type (wt)-HOTAIR (*p* < 0.05), while the luciferase activity in miR-221-3p mimic treated PD cell co-transfected with mutant (mut)-HOTAIR showed no difference (*p* > 0.05). In addition, the binding of miR-221-3p to HOTAIR was confirmed using RNA binding protein immunoprecipitation (RIP) assay, the results of which showed that the enrichment of miR-221-3p and HOTAIR was increased in cells treated with anti-Argonaute 2 (Ago2) compared to the cells treated with anti-IgG (*p* < 0.05) (Figure 3I). Therefore, it was suggested that HOTAIR bound to miR-221-3p.

### NPTX2 is a target gene of miR-221-3p

**Figure 4:**
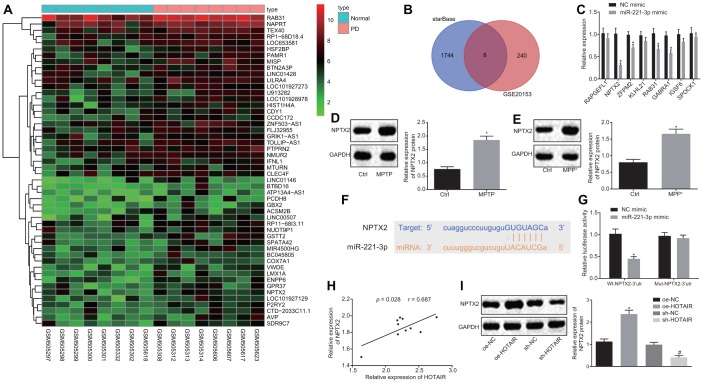
**miR-221-3p targets NPTX2.** The potential target genes of miR-221-3p predicted in the PD-related microarray data GSE20153, which was retrieved from the GEO database. (**A**) Differential analysis of the differentially expressed genes (DEGs) in normal and PD samples in GSE20153. (**B**) The expression patterns of the overlapped 8 genes measured by RT-qPCR, *p < 0.05 *vs*. the cells treated with NC mimic. (**C**) The expression patterns of NPTX2 in the SNc tissues of PD mouse models measured by Western blot analysis (n = 10), *p < 0.05 *vs*. mice injected with normal saline. (**D**) The expression patterns of NPTX2 in PD cell models measured using Western blot analysis, *p < 0.05 *vs*. the cells treated with PBS. (**E**) The complementary base paring diagram of miR-221-3p and NPTX2 predicted by an online website. (**F**) The binding of miR-221-3p to NPTX2 confirmed by dual-luciferase reporter gene assay, *p < 0.05 *vs*. cells treated with NC mimic. (**G**) Pearson’s correlation analysis of the expressions of HOTAIR and NPTX2 in the SNc tissues of PD mouse models. (**H**) The effect of over-expressed or down-regulated HOTAIR on the expression of NPTX2 measured by Western blot analysis, *p < 0.05 *vs*. cells treated with oe-NC, **#***p* < 0.05 *vs*. cells treated with sh-NC. (**I**) Data (mean ± standard deviation) between two groups or among multiple groups were analyzed using unpaired *t*-test. The experiment was repeated three times. miR-221-3p, microRNA-221-3p; NPTX2, neuronal pentraxin II; GEO, Gene Expression Omnibus; RT-qPCR, reverse transcription quantitative polymerase chain reaction; NC, negative control; SNc, substantia nigra compact; PD, Parkinson’s disease; PBS, phosphate buffered saline; wt, wild type; mut, mutase.

Furthermore, to elucidate the downstream regulatory target genes of miR-221-3p in PD, the PD-related microarray data GSE20153 was retrieved from the Gene Expression Omnibus (GEO) database. Differential analysis of the gene expressions in GSE20153 in normal samples and PD samples revealed that 248 genes were highly expressed in PD (Figure 4A). After that, the starBase database was employed to predict the potential target genes of miR-221-3p and the results were compared. There were a total of 8 intersecting genes which were screened from the two datasets (Figure 4B).

Subsequently, the expression patterns of the aforementioned eight genes were detected in PD cells, demonstrating that the expression of the NPTX2 gene was down-regulated when miR-221-3p was over-expressed (*p* < 0.05) (Figure 4C). Western blot analysis further revealed that NPTX2 was also highly expressed in the SNc tissues of PD mice and PD cells (all *p* < 0.05) (Figure 4D, 4E).

The binding sites between miR-221-3p and NPTX2 were retrieved using an online website (Figure 4F), which was further verified using a dual-luciferase reporter gene assay. Co-transfection of miR-221-3p mimic and wt-NPTX2-3’UTR resulted in decreased luciferase activity compared to PD cells treated with mimic NC (*p* < 0.05), but there was no difference in luciferase activity after co-transfection with miR-221-3p mimic and mut-NPTX2-3’UTR (*p* > 0.05) (Figure 4G). In addition, the expression of HOTAIR was found to be positively-correlated with that of NPTX2 in mouse PD models (Figure 4H), and similar trends were observed in PD cells (Figure 4I). Based on these results, it could be inferred that HOTAIR exerted its function in PD by elevating the expression of NPTX2 *via* binding to miR-221-3p.

### HOTAIR stimulates autophagy by elevating miR-221-3p dependent NPTX2 *in vitro*

**Figure 5:**
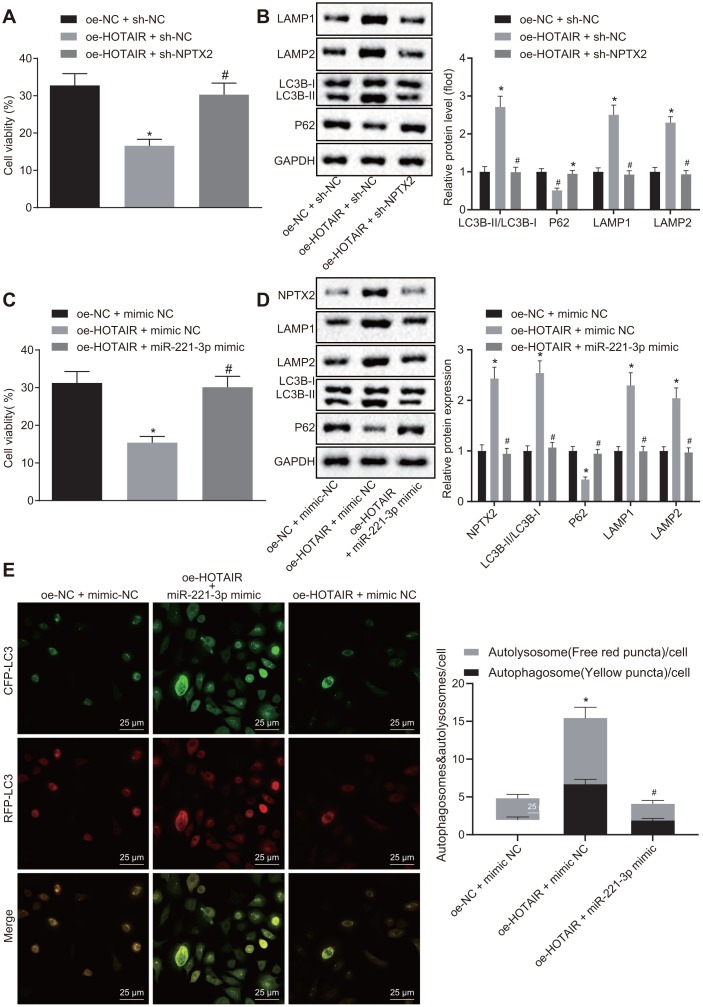
**HOTAIR elicits autophagy *via* miR-221-3p dependent NPTX2 elevation in PD cell models.** The viability of PD MN9D cells measured by the CCK8 assay, *p < 0.05 *vs*. PD MN9D cells treated with oe-HOTAIR + sh-NC. (**A**) The ratios of LC3B-I/LC3B-II and LAMP1/LAMP2 along with the P62 expression patterns in PD MN9D cell models measured by Western blot analysis as well as protein grayscale statistics, *p < 0.05 *vs*. PD MN9D cells treated with oe-NC + sh-NC, #*p* < 0.05 *vs*. PD MN9D cells treated with oe-HOTAIR + sh-NC. (**B**) The viability of PD MN9D cells measured by CCK8 assay, *p < 0.05 *vs*. PD MN9D cells treated with oe-NC + mimic NC, #*p* < 0.05 *vs*. PD MN9D cells treated with oe-HOTAIR + mimic NC. (**C**) The ratios of LC3B-I/LC3B-II and LAMP1/LAMP2 along with the P62 expression patterns in PD MN9D cell models measured by Western blot analysis as well as protein grayscale statistics, *p < 0.05 *vs*. PD MN9D cells treated with oe-NC + mimic NC, #*p* < 0.05 *vs*. PD MN9D cells treated with oe-HOTAIR + mimic NC. (**D**) Detection of autophagy levels in MN9D cells by co-localization analysis. (**E**) Data (mean ± standard deviation) among multiple groups were analyzed using one-way ANOVA and subjected to Tukey’s post-hoc test. The experiment was repeated three times. PD, Parkinson’s disease; HOTAIR, HOX transcript antisense intergenic RNA; miR-221-3p, microRNA-221-3p; NPTX2, neuronal pentraxin II; CCK-8, cell counting kit-8; NC, negative control.

Additionally, the potential effects of the HOTAIR/miR-221-3p/NPTX2 axis on autophagy in PD cells were evaluated. PD cells were treated with over-expression (oe)-NC + sh-NC, oe-HOTAIR + sh-NC and oe-HOTAIR + sh-NPTX2. Subsequently, CCK-8 assay was applied to measure the viability of PD cells, which illustrated that PD cells treated with oe-HOTAIR + sh-NC exhibited diminished viability compared to PD cells treated with oe-NC + sh-NC, while the viability of cells treated with oe-HOTAIR + sh-NPTX2 was much higher than those treated with oe-HOTAIR + sh-NC (all *p* < 0.05) (Figure 5A). In addition, the ratio of LC3B-I/LC3B-II and LAMP1/LAMP2 as well as P62 expression was detected using Western blot analysis. In comparison with oe-NC + sh-NC-treated cells, the ratio of LC3B-I/LC3B-II and LAMP1/LAMP2 was elevated but P62 expression was decreased in cells treated with over-expressed HOTAIR (*p* < 0.05). Meanwhile, the ratio of LC3B-I/LC3B-II and LAMP1/LAMP2 was decreased, while the expression of P62 was increased in PD cells treated with oe-HOTAIR + sh-NPTX2 in contrast to oe-HOTAIR + sh-NC treatment (all *p* < 0.05) (Figure 5B).

Subsequently, PD cells were treated with oe-NC + mimic NC, oe-HOTAIR + mimic NC and oe-HOTAIR + miR-221-3p mimic and their viabilities were assessed using CCK-8 assay. The viability of cells treated with oe-HOTAIR + mimic NC was reduced compared to those treated with oe-NC + mimic NC, while the viability of cells treated with oe-HOTAIR + miR-221-3p mimic was enhanced in contrast to oe-HOTAIR + mimic NC treatment (all *p* < 0.05) (Figure 5C). In addition, the expression patterns of NPTX2 and P62 as well as the ratio of LC3B-I/LC3B-II and LAMP1/LAMP2 were detected with Western blot analysis. It was found that the expressions of NPTX2 and the ratio of LC3B-I/LC3B-II and LAMP1/LAMP2 were elevated, whereas that of P62 was decreased in cells over-expressing HOTAIR compared to cells treated with oe-NC + mimic NC (*p* < 0.05). Meanwhile, compared with cells over-expressing HOTAIR, the expressions of NPTX2 and the ratio of LC3B-I/LC3B-II and LAMP1/LAMP2 were decreased, while the P62 expression was enhanced in cells co-transfected with oe-HOTAIR and miR-221-3p mimic (all *p* < 0.05) (Figure 5D). The above results verified that the HOTAIR/miR-221-3p/NPTX2 axis promoted autophagy in PD cell models.

### HOTAIR contributes to autophagy *via* miR-221-3p dependent NPTX2 elevation in vivo

**Figure 6:**
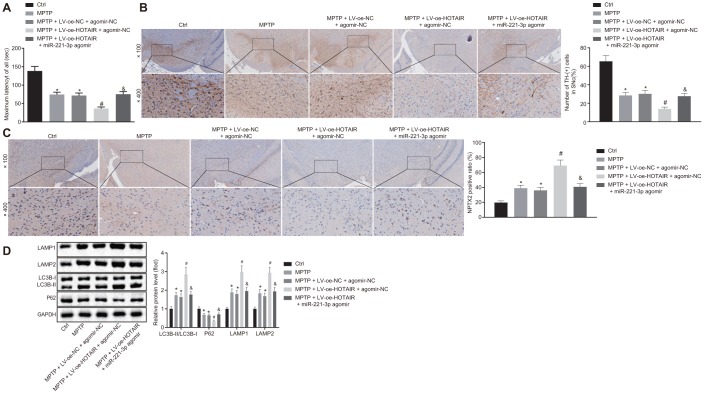
**HOTAIR induces autophagy *via* miR-221-3p dependent NPTX2 elevation in PD mouse models.** The retention time of PD mice in the rota rod test. (**A**) The number of TH positive cells in midbrain SNc tissues of PD mice tested by IHC. (**B**) The expression rate of NPTX2 in SNc tissues of PD mice detected by IHC. (**C**) The ratios of LC3B-I/LC3B-II and LAMP1/LAMP2 along with the P62 expression patterns in midbrain SNc tissues of PD mice measured by Western blot analysis (n = 10). (**D**) *p < 0.05 *vs*. PD mice injected with saline, #*p* < 0.05 *vs*. PD mice injected with MPTP + LV-oe-NC + agomir-NC, &*p* < 0.05 *vs*. PD mice injected with MPTP + LV-oe-HOTAIR + agomir-NC. Data (mean ± standard deviation) among multiple groups were analyzed using one-way ANOVA and subjected to Tukey’s post-hoc test. n = 10. PD, Parkinson’s disease; HOTAIR, HOX transcript antisense intergenic RNA; miR-221-3p, microRNA; NPTX2, neuronal pentraxin II; TH, T helper; SNc, substantia nigra compact; IHC, immunohistochemistry; MPTP, 1-methyl-4-phenyl-1,2,3,6-tetrahydropyrindine; NC, negative control.

To further explore the effects of the HOTAIR/miR-221-3p/ NPTX2 axis on autophagy in the PD mouse models, PD mice were initially injected with saline, MPTP, MPTP + LV-oe-NC + agomir-NC, MPTP + LV-oe-HOTAIR + agomir-NC and MPTP + LV-oe-HOTAIR + miR-221-3p agomir. The rota rod test was then performed to observe the retention time of the mice on the rotating rod. The results revealed that PD mice injected with MPTP and MPTP + LV-oe-NC + agomir-NC stayed on the rotating rod for shorter durations than PD mice injected with normal saline (*p* < 0.05); compared with PD mice injected with MPTP + LV-oe-NC + agomir-NC, PD mice injected with MPTP + LV-oe-HOTAIR + agomir-NC spent less time on the rotating rod, while PD mice injected with MPTP + LV-oe-HOTAIR + miR-221-3p agomir stayed longer than PD mice injected with MPTP + LV-oe-HOTAIR + agomir-NC (all *p* < 0.05) (Figure 6A).

Additionally, the number of TH positive cells in midbrain SNc tissues was counted by IHC. TH positive cells in the SNc tissues of PD mice injected with MPTP and MPTP + LV-oe-NC + agomir-NC were found to be decreased compared to those injected with normal saline (*p* < 0.05). Meanwhile, the number of TH positive cells was decreased in SNc tissues of PD mice treated by MPTP + LV-oe-HOTAIR + agomir-NC relative to those treated with MPTP + LV-oe-NC + agomir-NC (*p* < 0.05). In contrast, the mice injected with MPTP + LV-oe-HOTAIR + miR-221-3p agomir demonstrated increased number of TH positive cells compared to mice injected with MPTP + LV-oe-HOTAIR + agomir-NC (all *p* < 0.05) (Figure 6B).

Moreover, the positive expression rate of NPTX2 protein in SNc tissues of PD mice was determined using IHC, which highlighted up-regulated positive expression rate of NPTX2 protein in the SNc tissues of PD mice injected with MPTP and MPTP + LV-oe-NC + agomir-NC compared to those injected with normal saline (*p* < 0.05). Whereas, the positive expression rate of NPTX2 protein in the SNc tissues of PD mice injected with MPTP + LV-oe-HOTAIR + agomir-NC was much higher than that injected with MPTP + LV-oe-NC + agomir-NC, while the positive expression rate of NPTX2 in the SNc tissues of PD mice injected with MPTP + LV-oe-HOTAIR + miR-221-3p agomir was lower than that observed in PD mice injected with MPTP + LV-oe-HOTAIR + agomir-NC (all *p* < 0.05) (Figure 6C).

Lastly, the autophagy-related protein levels in PD mice were determined with Western blot analysis. As shown in Figure 6D, compared with PD mice injected with saline, the ratios of LC3B-I/LC3B-II and LAMP1/LAMP2 were increased, while the P62 protein expression was decreased in PD mice injected with MPTP or MPTP + LV-oe-NC + agomir-NC (*p* < 0.05). In PD mice injected with MPTP + LV-oe-HOTAIR + agomir-NC, the ratios of LC3B-I/LC3B-II and LAMP1/LAMP2 were increased and the P62 expression was reduced compared to the PD mice injected with MPTP + LV-oe-NC + agomir-NC (*p* < 0.05). Meanwhile, the ratios of LC3B-I/LC3B-II and LAMP1/LAMP2 were lower in PD mice injected with MPTP + LV-oe-HOTAIR + miR-221-3p agomir than that detected in PD mice injected with MPTP + LV-oe-HOTAIR + agomir-NC, while the P62 expression was higher (*p* < 0.05). These results verified that the HOTAIR/miR-221-3p/NPTX2 axis promoted autophagy in PD mice models.

## DISCUSSION

PD is a common neurological disorder characterized by dopaminergic neuron degeneration and death in the midbrain [26]. Research over the last few decades has formulated two chief mechanisms for this cell loss, one of which involves the aberrant accumulation of the protein α-synuclein bound to ubiquitin in the dopamine neurons, which ultimately forms insoluble inclusions known as Lewy bodies. The dissociative α-synuclein is toxic or even fatal to dopamine neurons [27, 28]. The other theory of PD entails dysfunction of lysosomal and proteasomal systems along with impaired mitochondrial activity [27, 29]. However, as diverse as they are, both of the two pathways are closely linked to the dysregulation of autophagy of neurons in PD. Interestingly, emerging studies have reported that several lncRNAs possess the ability to promote autophagy in MPTP-induced PD models, thus preventing PD progression [30, 31]. In the current study, we aimed to explore the ability of one such lncRNA HOTAIR to influence autophagy in PD, and further probed the underlying mechanisms involving miR-221-3p and NPTX2. Our collected findings indicated that silencing of HOTAIR could potentially inhibit autophagy of dopaminergic neurons *via* miR-221-3p-mediated down-regulation of the NPTX2 gene.

Initially, we uncovered that HOTAIR exerted high expressions in MPTP-induced mice PD models and MPP^+^-induced cell models of PD. HOTAIR is a multiply-functional lncRNA that has been reported to be expressed at high levels in various diseases [32]. In agreement with our findings, another study documented up-regulated levels of HOTAIR in the midbrain tissues of MTPT induced PD mice and in SH-SY5Y cells exposed to MPP^+^ [18]. Another key observation of the current study indicated that silencing of HOTAIR brought about a significant reduction in autophagy of dopaminergic neurons. The process of autophagy has been associated with many physiological and pathological processes [33]. Accumulation of autophagosomes occurs in postmortem brain samples from PD patients, which has been widely attributed to an induction of autophagy [34, 35]. Similar to our results, another study found that HOTAIR silencing inhibits autophagy and epithelial-mesenchymal transition *via* the Wnt signaling pathway in the Hela cell line [36]. Meanwhile, HOTAIR has also been shown to enhance cell autophagy through up-regulation of ATG3 and ATG7 in hepatocellular carcinoma [15]. In addition, a previous study demonstrated that silencing of HOTAIR decreased the drug resistance of non-small cell lung cancer cells to Crizotinib by curbing autophagy [23]. These previous findings and data support our position that silencing of HOTAIR markedly reduces autophagy of dopaminergic neurons.

Additionally, both *in vitro* and *in vivo* results from the current study revealed that silencing of HOTAIR disrupted autophagy of dopaminergic neurons through miR-221-3p dependent NPTX2 impairment. The NPTX2 gene has been closely correlated with neurological signs and disorders of the basal ganglia [37]. Moreover, another study documented elevated levels of NPTX2 in PD, which is in accordance with our findings [38]. Similarly, studies have also highlighted down-regulated levels of miR-221-3p as a biomarker for PD [20, 39]. However, the underlying mechanisms of miR-221-3p in regard to PD remain to be elucidated. Shedding some light on this regulation, our findings revealed that miR-221-3p specifically targeted the NPTX2 gene. A miR mechanism of NPTX2 down-regulation has also been previously detected in human brains following Alzheimer’s disease [21]. Meanwhile, we observed for the first time that HOTAIR can bind to miR-221-3p and subsequently inhibit its expression. Prior to this, HOTAIR was known to regulate a downstream target of miR-1 and disrupt the miR-1 expression [40]. In addition, another study predicted that HOTAIR binds to miR-130a and negatively-regulates its expression [41]. Whereas, the current study is the first to present a complete description of the HOTAIR/miR-221-3p/NPTX2 axis in dopamine cell autophagy. Similar to our findings, the HOTAIR/miR-20b-5p/ATG7 axis has also been shown to play a regulatory role in the autophagy of hepatic cells in hepatic ischemia/reperfusion injury [42]. In the present context of PD, HOTAIR has been previously highlighted to target miR-126-5p to promote disease progression through RAB3IP up-regulation [26]. Therefore, we contend that the HOTAIR/miR-221-3p/NPTX2 axis regulates autophagy of dopaminergic neurons in PD.

**Figure 7:**
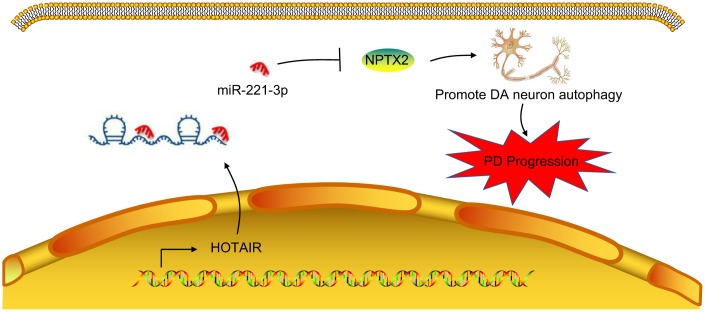
**A mechanism map depicting the role of the HOTAIR/miR-221-3p/NPTX2 axis in PD.** Inhibition of HOTAIR expression reduces autophagy of dopaminergic neurons through miR-221-3p-mediated down-regulation of NPTX2, thus retarding the onset and progression of PD.

Overall, our findings support that inhibition of HOTAIR reduces autophagy of dopaminergic neurons through miR-221-3p-mediated NPTX2 down-regulation in PD (Figure 7). These findings deepen our understanding of the molecular mechanisms in PD and highlight the discovery of a promising competitive new target for the treatment of PD. However, PD is conventionally held to be caused by failure of regular autophagy events. Our novel finding that aberrantly elevated autophagy of dopaminergic neurons can also be a factor in PD calls for new investigations to chart out the role of molecule interactions occurring in PD progression.

## MATERIALS AND METHODS

### Microarray-based gene expression profiling

The PD-related lncRNAs were retrieved from the LcnRNADisease database (http://www.cuilab.cn/lncrnadisease) and the localization of HOTAIR was predicted using the lncATLAS database (http://lncatlas.crg.eu/). In addition, the candidate downstream miRNAs regulated by HOTAIR were predicted using the lncRNA database (http://starbase.sysu.edu.cn/agoClipRNA.php?source=lncRNA), RNA22 (https://cm.jefferson.edu/rna22/), DIANA-LncBase (http://carolina.imis.athena-innovation.gr/diana_tools/web/index.php?r=lncbasev2/index), and miRcode (http://www.mircode.org/) databases. Additionally, the PD-related microarray dataset, GSE20153 was downloaded from the Gene Expression Omnibus database (https://www.ncbi.nlm.nih.gov/geo/). Subsequently, the “limma” package in the R language programming was applied for differential analysis of gene expressions in the normal samples and PD samples obtained from the GSE20153 dataset, while the “pheatmap” package was used to construct the gene expression heatmap. Furthermore, the potential downstream target gene of miR-221-3p was predicted using the miRNA-miRNA database (http://starbase.sysu.edu.cn/agoClipRNA.php?source=mRNA) from starBase.

### Establishment of MPTP-induced PD mouse models

A total of 70 male C57BL/6 mice (aged 8 weeks, weighing 25-30 g) were purchased from Experimental Animal Center of Dalian Medical University (Dalian, Liaoning, China), and reared and maintained under a 12-h light/dark cycle. Among these 70 mice, 10 mice were injected with normal saline and 10 mice were subcutaneously injected with MPTP (M0896; 30 mg/kg; Sigma-Aldrich Chemical Company, St Louis, MO, USA) once a day for a duration of one week [43]. The mice were then subjected to the rota rod test to evaluate abnormal motor functions. Afterwards, the mice were euthanized *via* intraperitoneal injection of excess sodium pentobarbital (150 mg/kg), and the brains were immediately excised, divided into two parts and preserved in liquid nitrogen for later experimentation. The remaining 50 mice were treated with LV and then subjected to PD model construction, and grouped into the following 5 groups (with 10 mice in each group): the LV-sh-NC group, the LV-sh-HOTAIR group, the LV-oe-NC + agomir-NC group, the LV-oe-HOTAIR + agomir-NC group and the LV-oe-HOTAIR + miR-221-3p agomir group.

### Rota rod test

A rotating rod with a diameter of 30 mm was applied and 5 mice were tested once at a time. The rotating speed was fixed at 12 rpm, and the mice were tested for a duration of 180 s and trained to keep themselves in the middle of the rod. The time spent by the mice on the rod was recorded 3 s after the rod was switched on. The mice were subjected to 3 consecutive tests every day and the longest time spent by each mouse on the rod was recorded. A double-blind method was employed for data collection and analysis for all *in vivo* assays.

### Lentivirus injection

Recombinant lentivirus LV-oe-HOTAIR and miR-221-3p agomir were purchased from the Hanbio Biotechnology Co., Ltd. (Shanghai, China). The lentiviruses were delivered into the midbrain SNc in the mice. A stereotaxic apparatus was applied to adjust the balance in the front-back and left-right directions according to the atlas of The Mouse Brain in Stereotaxic Coordinates. A 2 μL volume of lentiviruses (2 × 10^5^ TU/μL) or 10 mg/kg agomir (dissolved in 10 μL of normal saline) was injected into the mice at a speed of 0.25 μL/min. After two weeks, the mice were anaesthetized and then intraperitoneal injected with MPTP (M0896; 30 mg/kg; Sigma-Aldrich Chemical Company, St Louis, MO, USA) once a day to establish the mouse PD model. After 7 days of consecutive injections, the mice were euthanized and the SNc tissues were collected for biochemical analyses. Meanwhile, the mice injected with normal saline were transduced with aseptic saline solution (0.9%).

### Establishment of MPP^+^-provoked cell PD models

The dopaminergic neuronal cell line MN9D (American Type Culture Collection, Manassas, VA, USA) was cultured in high-glucose Dulbecco’s modified Eagle’s medium (DMEM) (Gibco, Carlsbad, CA, USA) containing 10% fetal bovine serum (FBS), 100 U/mL penicillin (Invitrogen Inc., Carlsbad, CA, USA) and 100 μg/mL streptomycin (Invitrogen Inc., Carlsbad, CA, USA) at 37°C with 5% CO_2_ in air. The cells were seeded in a 6-well plate. Upon reaching 80 - 90% confluence, the cells were transfected with the following plasmids in accordance with the instructions of Lipofectamine 2000 kits (Invitrogen Inc., Carlsbad, CA, USA): oe-NC, oe-HOTAIR, sh-NC, sh-HOTAIR, mimic NC, miR-221-3p mimic, inhibitor NC, miR-221-3p inhibitor, or sh-NPTX2 individually or together. After 24 h, the cells were manipulated with 100 μmol/L MPP^+^ (Sigma-Aldrich Chemical Company, St Louis, MO, USA) or phosphate buffered saline (PBS) for 24 h to induce the cell models of PD *in vitro*. Successfully modeled cells were selected for subsequent experimentation. During this grouping, a RAPA group, the autophagy positive control, did show an increase in the autophagy level. In addition, cells were treated with both MPP^+^ and 3-MA (an autophagy inhibitor) to confirm the inhibited autophagy level.

### mRFP-GFP-LC3 transfection and co-localization analysis

MN9D cells were seeded in CELLview TM glass bottom dishes (Scientific, USA) at a density of 1 × 10^3^ cells in 1 mL of serum containing DMEM until reaching 30% confluence. The cells were then transfected with the mRFP-GFP-LC3 plasmids and sh-NC or sh-HOTAIR for 24 h in DMEM supplemented with 10% FBS and 1% penicillin-streptomycin (P/S). Following DMEM removal, the cells were rinsed twice with PBS and then incubated for 24 h in serum containing DMEM. Next, the cells were incubated with 100 μmol/L MPP^+^ (Sigma-Aldrich Chemical Company, St Louis, MO, USA) for 24 h, and photographed using a laser scanning confocal microscope (LSM880, Carl Zeiss, Jena, Germany). The mRFP and GFP expressions in mRFP-GFP-LC3 tandem fluorescent protein adenovirus were used to track LC3. As GFP fluorescent protein is sensitive to acidity, the GFP fluorescence was quenched following the fusion of autophagosome and lysosome. Therefore, weakened GFP signal could indicate the fusion of autophagosome and lysosome to form an autolysosome. When red and green fluorescence merged, the yellow spots represented autophagosomes and red spots represented autolysosomes. When autophagy occurred, there were more red spots than yellow spots, and when the fusion of autophagosomes-lysosomes was damaged, the yellow spots were dominant. Finally, 6 non-overlapping fields were observed, with at least 20 cells counted in each group.

### CCK-8 assay

MN9D cells were seeded in a 96-well plate, and incubated with 100 μmol/L MPP^+^ (Sigma-Aldrich Chemical Company, St Louis, MO, USA) for 96 h. Afterwards, the cell viability was measured using CCK-8 kits (Dojindo, Kumamoto, Japan). The proliferation rate of PBS-treated cells was normalized to 1 and expressed as mean ± standard deviation.

### RT-qPCR

Total RNA content was extracted using RNeasy Mini kits (Qiagen, Valencia, CA, USA) and then reverse transcribed into complementary DNA (cDNA) using reverse transcription kits (RR047A, Takara, Tokyo, Japan). Determination of miRNA was conducted by the tailing reaction and polyadenylation of isolated RNA using the NCode™ miRNA First-Strand cDNA Synthesis Kit (MIRC10, Invitrogen, Carlsbad, California, USA). Subsequently, the SYBR Premix EX Taq (RR420A, Takara, Tokyo, Japan) was applied for sample loading and RT-qPCR was performed using a real-time fluorescence quantitative PCR instrument (ABI7500, ABI, Foster City, CA, USA). Three duplicate wells were set for each sample. The reverse primers of the miRNAs were the general primer provided by the NCode^TM^ miRNA first strand cDNA synthesized kits and the remaining primers were synthesized by Sangon Biotech (Shanghai) Co., Ltd. (Shanghai, China) (Table 1). U6 was regarded as the internal reference for miRNAs, while glyceraldehyde-3-phosphate dehydrogenase (GAPDH) was used for lncRNAs and coding genes. The relative expressions were calculated using the 2^-ΔΔCt^ method.

**Table 1:** Primer sequences for reverse transcription quantitative polymerase chain reaction.

Gene	Primer sequence
MALAT1	F: 5′-GTAGGTTAAGTTGACGGCCGTTA-3′
	R: 5′-ATCTTCCCTGTTTCCAACTCATG-3′
HOTAIR	F: 5′-GGCGGATGCAAGTTAATAAAAC-3′
	R: 5′-TACGCCTGAGTGTTCACGAG-3′
miR-17-5p	F: 5′-CAAAGTGCTTACAGTGCAGGTAG-3′
miR-20b-5p	F: 5′-CAAAGTGCTCATAGTGCAGGTAG-3′
miR-221-3p	F: 5′-AGCTACATTGTCTGCTGGGTTTC-3′
miR-301b-3p	F: 5′-CAGTGCAATGGTATTGTCAAAGC-3′
RAPGEFL1	F: 5′-CCTGGGCCGATAGGATCTG-3′
	R: 5′-GGAATGGGTAAGAACAATGTCGT-3′
NPTX2	F: 5′-CTCAAGGACCGCTTGGAGAG-3′
	R: 5′-GGTCTCATTATGAAGCAGGGAC-3′
ZFPM2	F: 5′-AAACCCCGGCAGATCAAAC-3′
	R: 5′-GCTCAGATTTTCGGGCTCAAA-3′
KLHL21	F: 5′-CCGCTAGTCCCTACTTCCG-3′
	R: 5′-CTCTGCCGGTGTAGCTGAA-3′
RAB31	F: 5′-GACACGGGGGTTGGGAAATC-3′
	R: 5′-ACAAGGCACGGTTTTGGTCA-3′
GABRA1	F: 5′-TGAGCACACTGTCGGGAAGA-3′
	R: 5′-CAGCAGTCGGTCCAAAATTCT-3′
IGSF6	F: 5′-CCTGATCCTTTTCCAAGTCGG-3′
	R: 5′-ACCGTAGTCCACTTCTAGGTAAC-3′
SPOCK1	F: 5′-ATGCAGCCCGCACAAAGTAT-3′
	R: 5′-CACTTAACCAGATTGGAAGGTCC-3′
GAPDH	F: 5′-TTCACCACCATGGAGAAGGC-3′
	R: 5′-GGCATGGACTGTGGTCATGA-3′
U6	F: 5′-CTCGCTTCGGCAGCACA-3′
	R: 5′-AACGCTTCACGAATTTGCGT-3′

Notes: F, forward; R, reverse; MALAT1, metastasis associated in lung adenocarcinoma transcript 1; HOTAIR, HOX transcript antisense intergenic RNA; miR, microRNA; RAPGEFL1, rap guanine nucleotide exchange factor like 1; NPTX2, Neuronal pentraxin II; ZFPM2, Zinc finger protein, FOG2 family member 2; KLHL21, Kelch-like Protein 21; GABRA1, GABA-A receptor alpha 1 subunit gene; IGSF6, immunoglobulin superfamily 6; GAPDH, glyceraldehyde-3-phosphate dehydrogenase.

### Fish

The subcellular location of HOTAIR was detected using FISH according to the protocols of Ribo^TM^ lncRNA FISH Probe Mix (Red) (C10920, Guangzhou RiboBio Co., Ltd., Guangzhou, Guangdong, China). In brief, the cell slide was placed in a 24-well culture plate and the cells were plated at a density of 6 × 10^4^ cells/well. When the cell confluence reached 60 - 70%, the cells were fixed with 1 mL of 4% paraformaldehyde for 10 min, added with 1 mL of pre-cooled PBS containing 0.5% Triton X-100 at 4°C for 5 min, and then blocked with 200 μL of pre-hybridization solution at 37°C for 30 min. Upon discarding the pre-hybridization solution, cells in each well were hybridized overnight with hybridization solution containing probes (anti-HOTAIR nucleotide probe; Wuhan GeneCreate Biological Engineering Co., Ltd., Wuhan, China). After being washed with washing lotion I, II, III, and 1× PBS at 42°C, the cells were stained with 4’,6-diamidino-2-phenylindole (1 : 800), mounted with nail polish, and observed under a fluorescence microscope (Olympus, Optical Co., Ltd, Tokyo, Japan).

### RIP assay

MN9D cells were incubated with RIP Lysis Buffer (N653-100 mL, Shanghai Haoran Bio Technologies Co., Ltd., Shanghai, China) and lysed on ice for 5 min. The lysate was then mixed with 50 μL of magnetic beads, added with 0.5 mL of RIP Wash Buffer (EHJ-BVIS08102, Xiamen Huijia Biotechnology Co., Ltd., Xiamen, China), and placed on a magnetic separator for bead aggregation. After that, the beads were incubated with 5 μg of Ago2 antibody (P10502500, Otwo Biotech [Shenzhen] Inc., Shenzhen, Guangdong, China) for 30 min. The antibody was replaced with normal mouse immunoglobulin G (IgG) in the control group. After discarding the supernatant, the beads were washed with 0.5 mL of RIP Wash Buffer. Next, the beads-antibody complex was mixed with 900 μL of RIP Immunoprecipitation Buffer (P10403138, Otwo Biotech [Shenzhen] Inc., Shenzhen, Guangdong, China). The complex was incubated overnight at 4°C, washed with 0.5 mL of RIP Wash Buffer, and then incubated with 150 μL of proteinase K buffer at 55°C for 30 min. Lastly, the RNA was subsequently isolated and determined using RT-qPCR.

### Western blot analysis

The mice were euthanized following behavioral tests and the whole brain was excised, after which the SNc tissues were dissected under a stereomicroscope. Total protein content from the tissues or cells was extracted using radioimmunoprecipitation assay (RIPA) lysis buffer, and the protein concentration was measured using a bicinchoninic acid kit. Next, the protein was separated by sodium dodecyl sulfate-polyacrylamide gel electrophoresis (SDS-PAGE) and then transferred onto polyvinylidene fluoride membranes. The membrane was then blocked using 5% skimmed milk at room temperature for 1 h and incubated overnight at 4°C with the following primary antibodies, purchased from Abcam Inc. (Cambridge, UK): rabbit polyclonal antibody to NPTX2 (ab69858, dilution ratio of 1 : 1000), rabbit polyclonal antibody to LC3B (ab51520, dilution ratio of 1 : 3000), rabbit polyclonal antibody to LAMP1 (ab24170, dilution ratio of 1 : 2000), LAMP2 (ab13524, dilution ratio of 1 : 2000), rabbit polyclonal antibody to P62 (ab91526, dilution ratio of 1 : 1000), and rabbit monoclonal antibody to GAPDH (ab181603, dilution ratio of 1 : 10000) as the loading control. After a rinse with Tris-buffered saline Tween-20 (TBST), the membrane was incubated with horseradish peroxidase (HRP)-labeled goat anti-rabbit IgG H&L (ab97051, dilution ratio of 1 : 2000, Abcam Inc., Cambridge, UK) for 1 h. The immunocomplexes on the membrane were visualized using an enhanced chemiluminescence (ECL) reagent and the Bio-Rad imaging analysis system (Bio-Rad, Hercules, CA, USA). The band intensities were quantified using the Quantity One v4.6.2 software, and the ratio of the gray value of the target band to GAPDH was representative of the relative protein expressions.

### Dual-luciferase reporter gene assay

The wt and mut reporter plasmids of HOTAIR (wt-HOTAIR; mut-HOTAIR) and NPTX2 (wt-NPTX2 and mut-NPTX2) were designed by Shanghai GenePharma Co., Ltd. (Shanghai, China). NC mimic or miR-221-3p mimic was co-transfected with wt-HOTAIR, mut-HOTAIR, wt-NPTX2 or mut-NPTX2 into the MN9D cells. After 24 h, the cells were treated with 100 μmol/L MPP^+^ (Sigma-Aldrich Chemical Company, St Louis, MO, USA) or PBS for 24 h and then lysed. The luciferase activity of the target reporter gene was analyzed according to the instructions of the luciferase detection kit (K801-200, BioVision, Milpitas, CA, USA).

### IHC

The mice were anesthetized by intraperitoneal injection of 10% uratan and 2% chloral hydrate (0.1 mL/10 g). Cardiac perfusion was then performed with 0.9% normal saline and 4% paraformaldehyde solution respectively. The whole brain was excised, paraffin-embedded and sectioned. Four brain sections were taken from each group and stained. The SNc tissue sections of PD mice were baked at 60°C for 1 h, deparaffinized using routine xylene, and hydrated with gradient ethanol, followed by antigen retrieval under high pressure for 2 min. After that, the sections were incubated with the primary rabbit polyclonal antibody to TH (ab112, dilution ratio of 1 : 200, Abcam Inc., Cambridge, UK) or rabbit anti-mouse NPTX2 (ab69858, dilution ratio of 1 : 1000, Abcam Inc., Cambridge, UK) at 37°C for 2 h. The detailed steps were performed according to previously published protocols [44]. The number of positive cells was counted in 5 high-power randomly selected visual fields from each section.

### Statistical analysis

All data were processed using the SPSS 22.0 software (IBM Corp. Armonk, NY, USA). Measurement data were expressed as mean ± standard deviation. The paired-design data conforming to normal distribution and homogeneity of variance between two groups were analyzed using the paired *t*-test. The unpaired-design data conforming to normal distribution and homogeneity of variance between two groups were analyzed using unpaired *t*-test. Data among multiple groups were tested using one-way analysis of variance (ANOVA), followed by Tukey’s post-hoc test. Pearson’s correlation analysis was employed to analyze the correlation between HOTAIR and NPTX2 expression in SNc tissues in MPTP-induced PD mouse models. A value of *p* < 0.05 was indicative of statistical significance.

### Ethics statement

Study protocols in the current study were approved by the Experimental Animal Ethics Committee of the Second Hospital of Dalian Medical University. All animal experimentation strictly adhered to principles aiming to minimize the pain, suffering and discomfort to experimental animals.

## Supplementary Material

Supplementary Table 1
