# Supplementary material for: HOTAIR drives autophagy in midbrain dopaminergic neurons in the substantia nigra compacta in a mouse model of Parkinson’s disease by elevating NPTX2 via miR-221-3p binding
Source: Aging (Albany NY). 2020 May 12;12(9):7660–78. doi: 10.18632/aging.103028 (PMC7244061; doi:10.18632/aging.103028)
Supplement: Supplementary Table 1 [file aging-12-103028-s001..pdf]

SUPPLEMENTARY TABLE

Supplementary Table 1. PD-related lncRNAs retrieved from LncRNADisease database.

LncRNA name	Disease name	Dysfunction type	Species	GenBank	Reference
MALAT1	Parkinson’s disease	Regulation	Human	NR_002819	27470562
UCHL1-AS1	Parkinson’s disease	Regulation	Human	NR_102709	27338628
AK021630	Parkinson’s disease	Regulation	Human	N/A	27151187
HOTAIR	Parkinson’s disease	Regulation	Human	NR_003716	26979073

Notes: lncRNAs, long non-coding RNAs; PD, Parkinson’s disease; MALAT1, metastasis associated in lung adenocarcinoma transcript 1; UCHL1, ubiquitin C-terminal hydrolase 1; HOTAIR, HOX transcript antisense intergenic RNA.
